# Free Amino Acids in Human Milk: A Potential Role for Glutamine and Glutamate in the Protection Against Neonatal Allergies and Infections

**DOI:** 10.3389/fimmu.2020.01007

**Published:** 2020-05-28

**Authors:** Joris H. J. van Sadelhoff, Selma P. Wiertsema, Johan Garssen, Astrid Hogenkamp

**Affiliations:** ^1^Division of Pharmacology, Utrecht Institute for Pharmaceutical Sciences, Faculty of Science, Utrecht University, Utrecht, Netherlands; ^2^Danone Nutricia Research, Utrecht, Netherlands

**Keywords:** human milk, free amino acids, glutamine, glutamate, neonates, immune development, allergies, infections

## Abstract

Breastfeeding is indicated to support neonatal immune development and to protect against neonatal infections and allergies. Human milk composition is widely studied in relation to these unique abilities, which has led to the identification of various immunomodulating components in human milk, including various bioactive proteins. In addition to proteins, human milk contains free amino acids (FAAs), which have not been well-studied. Of those, the FAAs glutamate and glutamine are by far the most abundant. Levels of these FAAs in human milk sharply increase during the first months of lactation, in contrast to most other FAAs. These unique dynamics are globally consistent, suggesting that their levels in human milk are tightly regulated throughout lactation and, consequently, that they might have specific roles in the developing neonate. Interestingly, free glutamine and glutamate are reported to exhibit immunomodulating capacities, indicating that these FAAs could contribute to neonatal immune development and to the unique protective effects of breastfeeding. This review describes the current understanding of the FAA composition in human milk. Moreover, it provides an overview of the effects of free glutamine and glutamate on immune parameters relevant for allergic sensitization and infections in early life. The data reviewed provide rationale to study the role of free glutamine and glutamate in human milk in the protection against neonatal allergies and infections.

## Introduction

Human milk is widely recognized as the best source of infant nutrition. It provides the infant with a highly diverse mix of nutrients that supports optimal development. The health benefits of human milk, however, go beyond that of providing nutrients. An increasing body of evidence suggests that human milk provides the neonate with a protection against a variety of immune-related conditions. For example, it is shown consistently that infants who were exclusively breastfed were less likely to develop respiratory and gastrointestinal infections than infants who fully or partially received an infant milk formula (1–5). This protective effect of breastfeeding against infections may extend well beyond infancy and is indicated to be enhanced upon prolonged breastfeeding (6, 7). Furthermore, studies have demonstrated that exclusive breastfeeding protects against various allergic diseases, including atopic dermatitis (8, 9), asthma (9–11) and food allergy (12–15), especially if there is a family history of allergic disease (16). For cow's milk allergy, which is one of the most common food allergies in infants, the incidence rate is reported to be up to seven times lower in exclusively breastfed infants, compared to infants fully or partially fed an infant milk formula (17–19). These unique protective capacities of human milk have driven scientific research into the underlying mechanisms in the past decades (15, 20, 21).

At birth, the immune system is immature (22). Compared to adults, the neonatal immune system is characterized by diminished innate effector cell functions, suppressed T-helper 1 (T_H_1) immune responses and skewed T cell responses to antigens toward T-helper 2 (T_H_2) immunity. These characteristics correlate with an increased susceptibility to infections and allergies in the neonatal period (23, 24). This susceptibility is further enhanced by an immature intestinal barrier function and an incomplete intestinal microbial colonization at birth (23). Various factors in human milk have been identified that could support the development of these immune functions, and thus may contribute to the protection against infections and allergies. For instance, human milk contains immunoglobulin A (IgA) antibodies, which confer protection against pathogens and are reported to induce tolerance to food allergens (25, 26). Moreover, various bioactive oligosaccharides, fatty acids and proteins have been identified in human milk that are capable of modulating immune responses directly, e.g., by regulating immune responses to pathogens (27–29), and indirectly, e.g., by shaping the gut microbiome (29–32). In addition to proteins, human milk also contains protein-unbound, free amino acids (FAAs). Accumulating evidence indicates that certain FAAs are bioactive, and more specifically have immunomodulating capacities (33, 34). Hence, FAAs in human milk may play an active part in an optimal immune development of the infant. However, whereas research on physiological functions of FAAs has made significant progress in recent years, FAAs are typically overlooked in human milk research.

Of the total content of amino acids (AAs) in human milk, 5–10% is present in free form. The FAAs glutamate and glutamine are by far the most abundant, both in absolute sense and relative to their protein-bound form, together comprising almost 70% of all FAAs present in human milk (35). Their levels display unique and consistent patterns over lactation, suggesting that secretion of these FAAs in human milk is a regulated process (35, 36). Interestingly, these structurally related FAAs have been widely associated with immunomodulation, including the modulation of immune mechanisms relevant for the development of allergies and infections. This review aims to describe the current understanding of the FAA composition in human milk, and to provide an overview of the effects of the FAAs glutamine and glutamate on immune parameters relevant for allergic diseases and infections in early life. Ultimately, a better understanding of the composition of FAAs in human milk and their immunomodulating capacities may contribute to the development of new avenues in the prevention of allergies and infectious diseases in infancy.

## Amino Acids in Human Milk: Protein-Bound and Free Amino Acids

It is well known that protein quality and quantity are key aspects of the nutritional value of human milk. The total amino acid (TAA) composition of human milk, including protein-bound AAs and FAAs, is used to evaluate the quantity and the quality of the milk proteins and hence is well characterized (36, 37). However, many studies only report the TAA composition and do not distinguish between protein-bound and FAAs. As a result, data on FAAs in human milk are relatively limited.

FAAs in human milk have been reported to account for ~5–10% of the TAA content (35, 36). Despite their low abundance relative to protein-bound AA levels, the relevance of FAAs in human milk should not be underestimated. Their levels are approximately 100 times higher than the 0.05% FAA pool in tissues (38) and up to 30 times higher than the FAA levels in plasma of infants (39). Moreover, FAAs in human milk contribute significantly to the initial changes in plasma levels of FAAs following a feed (40, 41) and are indicated to be more readily absorbed (42–44), appear sooner in the circulation and thus might reach peripheral organs and tissues faster than protein-derived AAs. Indeed, differences in plasma FAA levels were observed between infants receiving an infant milk formula containing FAAs and infants receiving an equivalent portion of AAs in the form of intact protein, suggesting differences in absorption kinetics between FAAs and protein-derived AAs (45–47). In contrast to their protein-bound counterpart, FAAs can interact with specific receptors present on a wide variety of cells in various parts of the body, including the intestines, where they can activate specific intracellular pathways and confer physiological effects (34, 48).

While human milk directly supplies infants with FAAs, human milk proteins could also provide the infant with FAAs via proteolysis in the neonatal gastrointestinal tract. However, the contribution of proteolysis of human milk proteins to the FAA supply of infants might be relatively low, as (complete) proteolysis of these proteins in infants is shown to occur to a minimal extent (49–52). Factors contributing to the limited proteolysis of human milk proteins are the relatively low output of pepsin and gastric enzymes observed in infants, the relatively high gastric postprandial pH which leaves proteases largely inactive, as well as the high degree of glycosylation of these proteins (50). Accordingly, it has been argued that the availability of FAAs in the upper region of the gastrointestinal tract, including the upper parts of the small intestine, is almost entirely dependent on the dietary FAA content (48).

The unique abilities of FAAs compared to protein-bound AAs and the relatively inefficient proteolytic capacity of neonates underline the importance of understanding the FAA composition in human milk, separate from the TAA composition.

### The FAA Composition in Human Milk is Dynamic and Seemingly Regulated

The composition of human milk is known to be dynamic over the course of lactation. The total protein content has been consistently shown to decrease in the first 3 months of lactation (35, 36). It is argued that this decrease correlates with the infant's protein requirements for growth and that it prevents overfeeding, as milk volume intake increases during this period (53, 54). Not surprisingly, similar dynamics are found for the protein-bound AA content in human milk. For each individual AA the protein-bound form decreases to a highly similar extent during lactation, indicating that the dynamics of protein-bound AAs in human milk during lactation are not AA-specific (35). In contrast, levels of FAAs in human milk display dynamics during lactation that are highly AA-specific: whereas levels of some FAAs decrease in the first 3 months of lactation, others remain stable or sharply increase (35, 36). Remarkably, these FAA dynamics during lactation are consistent in studies across various ethnic groups and geographical locations, indicating that these dynamics are globally consistent and thus seemingly regulated (35, 36, 55, 56).

The underlying mechanisms regulating the dynamics of FAA levels in human milk are poorly understood. Cells of the mammary gland secrete proteases and anti-proteases into human milk that together regulate the cleavage of specific AAs from human milk proteins, generating FAAs and peptides (57). Thus, it can be hypothesized that temporal changes in net proteolytic activity in human milk contribute to the FAA dynamics, although this is unlikely as levels of all major human milk proteases and anti-proteases decrease during lactation, along with levels of their substrates (50, 58). Mammary gland cells can also directly secrete FAAs into human milk via AA transporters present on their cell membranes. Interestingly, animal studies have shown that the expression of certain AA transporters in the mammary gland increases with progressing lactation, whereas that of others remains unchanged (59–62). These expression dynamics throughout lactation appear to be tightly regulated by multiple intracellular signaling pathways (63). Thus, it can be speculated that the dynamic expression of AA transporters on mammary gland cells along lactation contributes to the FAA dynamics in human milk.

To better understand the mechanisms underlying the secretion of FAAs in human milk, several studies examined the influence of maternal characteristics on the FAA composition in human milk. Whereas, FAA levels seem to be independent of the mothers' age (64), maternal body-mass index is reported to slightly influence levels of several FAAs (65, 66). Mechanisms underlying this effect are not known, but may involve the hormone prolactin, as prolactin is involved in regulating FAA transport in the mammary gland and levels of prolactin associate with maternal body-mass index (67–69). Studies investigating the effect of maternal diet on the AA composition in human milk indicate that the TAA composition is largely independent of the AA composition of the diet (70, 71). For FAAs, this relation remains to be examined in humans. However, studies across different geographical locations where different diets are consumed show largely similar levels and ratios of FAAs in human milk, suggesting that maternal diet is not of major influence (35, 36, 55, 56). This is supported by the finding that oral supplementation of a single load of glutamate (6g) in healthy lactating women did not alter levels of any of the FAAs in their breastmilk (72). Moreover, several studies reported that there was no association between maternal plasma levels of FAAs and the FAA levels in human milk (73, 74). In fact, some FAAs were 1- to 15-fold higher in plasma compared to milk, whereas levels of free glutamate were 40-fold higher in milk than in plasma.

All together, these findings indicate that selective FAA transport occurs in mammary tissues during lactation and that levels of FAAs in human milk might be highly regulated throughout lactation.

### Correlations of FAAs in Human Milk With Lactation Stage, Gestational Age and Infant Anthropometrics: A Special Role for Free Glutamine and Glutamate?

The FAAs glutamine, glutamate, glycine, serine and alanine in human milk have consistently been shown to increase in the first 3 months of lactation, whereas the levels of most other FAAs remain relatively stable along lactation (35, 36, 55, 56). Of these, glutamate is by far the most abundant, accounting for more than 50% of the total FAA content at any stage of lactation. In addition, glutamate shows the highest absolute increase in concentration along lactation, increasing from ~1.25 to 1.75 mM from month 1 to 6 of lactation (35). Glutamine, the second-most abundant FAA, shows the highest relative increase in concentration, increasing almost 350% from month 1 to 6 of lactation and reaching a concentration of up to 0.6 mM (35, 64, 75). In addition to the stage of lactation, the gestational age of the infant has also been reported to be a determinant of the free glutamine levels in human milk. A meta-analysis has shown that free glutamine levels in milk for preterm infants are almost three times lower than those observed in milk for term infants in the first month of lactation (36). Levels of all other FAAs were similar in preterm and term human milk samples, indicating that this difference was AA-specific.

Studies investigating associations of FAAs with infant anthropometrics are scarce but do report consistent findings. It was recently reported that free glutamate levels in human milk were significantly higher for term infants that had faster weight gain (76). Moreover, glutamine levels also tended to be higher for fast growing children. Consistent with these findings, another study reported a positive association between free glutamine levels in human milk and infant length at 4 months of age (65). These findings are in line with studies indicating that milk for boys tends to have higher levels of free glutamine and glutamate than milk for girls in the first 3 to 4 months of lactation (35, 76), as boys are known to gain more weight and length than girls in this time period (77).

The finding that levels of free glutamine and glutamate in human milk are relatively high, display unique dynamics along lactation, and are associated with infant anthropometrics urges the need to understand the functions that these FAAs could have during infant development.

## The Diversity in Physiological Functions of Free Glutamine and Glutamate

**Figure 1:**
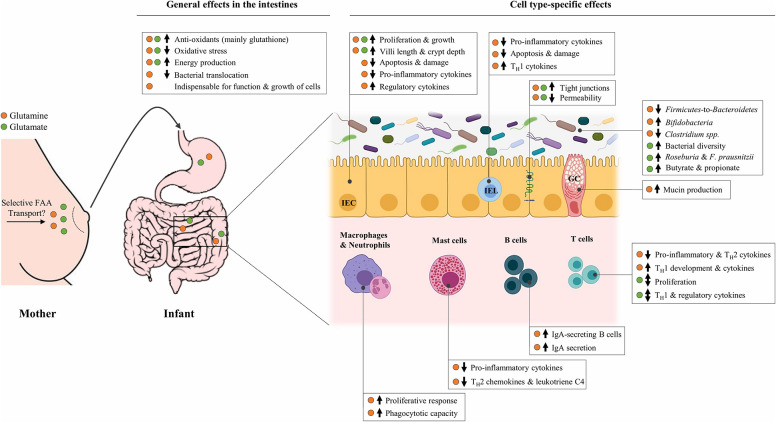
Overview of the potential effects of free glutamine and glutamate, selectively secreted in human milk by mammary gland cells, in the developing infant gut. The **↑** and **↓** indicate an upregulation and downregulation, respectively, of the corresponding target following *in vitro* and/or *in vivo* supplementation with glutamine (•) or glutamate (•). Effects are limited to those that are relevant in the context of allergic sensitization and infections. FAA, Free amino acid; IEC, Intestinal epithelial cell; IEL, Intraepithelial lymphocyte; GC, Goblet cell; T_H_1, T-helper 1 cell; T_H_2, T-helper 2 cell; IgA, Immunoglobulin A; *F. prausnitzii, Faecalibacterium prausnitzii*.

In the last decade, it has been recognized that glutamine and glutamate are essential AAs at key times in life, including the neonatal period when rapid growth occurs (78, 79). Although these two FAAs are structurally related, they appear to be different in terms of absorption by the infant. Whereas dietary glutamine supplementation in infants leads to higher plasma levels of this AA (80, 81), plasma levels of glutamate are largely unaffected by dietary glutamate (82, 83). This suggests that free glutamate in human milk is almost entirely used by splanchnic tissues, limiting its availability for other tissues, whereas glutamine might also exert direct effects elsewhere in the body. Despite these differences, most of the dietary glutamine and glutamate provided to neonates is consistently shown to be used by the intestines (84, 85). The intestines do not only form a physical barrier to protect against pathogens but are also home to the largest immune organ of the body: the gut-associated lymphoid tissue (GALT). This may explain why glutamine and glutamate are associated with a wide range of physiological functions, ranging from energy provision to cells to more specific immunomodulating functions, many of which could be relevant in the context of the prevention of neonatal allergies and infections. Figure 1 provides a summary of the demonstrated effects of free glutamine and glutamate in (developing) intestinal tissues, which are described in detail below.

### Metabolism of Glutamine and Glutamate in Intestinal Epithelial Cells and Immune Cells: Their Function as Energy Substrate and Protein Precursors

It is well-established that glutamine and glutamate are important energy substrates for intestinal epithelial cells (IECs) and immune cells, especially during periods of rapid growth (86). In fact, studies in young animals and infants have shown that approximately half of the dietary glutamate and glutamine is oxidized by intestinal and immune cells, ultimately leading to the generation of energy for the cells to adequately function and grow (87). Intestinal cells can convert glutamine into glutamate, which is crucial for the usage of glutamine for energy purposes (88). Whereas, human intestinal cells can also convert glutamate into glutamine, this process is limited due to the low glutamine synthetase activity in the small intestine (89, 90). In the neonatal period, this ability may be further limited as studies in young rats demonstrated that glutamine synthetase activity is particularly low in the pre-weaning period (91, 92). Remarkably, IECs as well as immune cells cannot function properly without the availability of exogenous glutamine (93). This, combined with their limited capacity to synthetize glutamine suggests that adequate functioning of these cells in the neonatal period might be partially dependent on dietary-derived glutamine.

Besides serving as energy substrates, free glutamine and glutamate are both specific precursors for glutathione, which is the main antioxidant in IECs and immune cells and critical for the prevention of cellular damage caused by pro-oxidants (94). An imbalance in pro- and antioxidants, known as oxidative stress, stimulates inflammatory responses that can lead to the development and maintenance of allergic disorders (95, 96). Hence, antioxidants like glutathione are considered as preventive or treatment strategy for food allergies (97). It has been reported that both dietary glutamine and dietary glutamate enhance glutathione production and, possibly as a result, reduce oxidative stress in the intestines of weaning piglets (98, 99). In addition, glutamine, but not glutamate, is an important specific precursor for the synthesis of mucins, which are critical for the defense against infections and are suggested to protect against allergic sensitization (100–103). Accordingly, oral glutamine supplementation has been shown to enhance mucin synthesis and to increase the number of mucin-secreting goblet cells in the small intestine of weaned piglets (104).

### Effects of Free Glutamine and Glutamate on Intestinal Growth and Barrier Function

In the rapidly growing neonate where the intestines are not yet fully developed, it is crucial to achieve and maintain rapid growth of IECs. Moreover, it is well-established that intestinal barrier function is a crucial factor in the protection against allergies and infections, by preventing allergen and bacterial translocation from the gut lumen into the immune cell-populated lamina propria and mesenteric lymph nodes (105–107). In neonates where intestinal barrier function is immature, proper availability of nutrients that contribute to the growth of IECs and maturation of the intestinal barrier is critical to support this protective effect. Interestingly, free glutamine and glutamate have been shown consistently to influence these processes, by various mechanisms which are further explained in the following sections.

#### Impact of Glutamine on Intestinal Functions

Glutamine is by far the most widely examined AA in relation to growth and function of IECs. This FAA is known to stimulate IEC proliferation in a variety of ways, as demonstrated in various neonatal IEC lines *in vitro*. For instance, glutamine dose-dependently enhanced cell proliferation and differentiation of neonatal porcine and rat IECs, through activating multiple mitogen-activated protein kinases (MAPKs) (108–110). Moreover, studies in neonatal porcine and adult human IEC lines have indicated that glutamine also promotes growth through augmenting the effects of growth factors, including insulin-like growth factor 1 and epidermal growth factor (108, 111–113). In addition to promoting growth, glutamine has been reported to dose-dependently protect against inflammation-, endotoxin- and oxidant-induced cell death and damage in these IEC lines (114–116). Remarkably, glutamine completely blocked inflammation-induced apoptosis in the adult human epithelial cell line HT-29 when supplied at 0.5 mM, a concentration similar to that of free glutamine in human milk (115).

Multiple lines of evidence indicate that glutamine also specifically stimulates intestinal barrier function. For instance, *in vitro* studies with neonatal porcine and human adult IEC lines have revealed that glutamine restriction reduces the expression of the major tight junction proteins, including claudin and occludin proteins, which are vital for intestinal barrier function (110, 117, 118). This was accompanied by a reduced distribution of these proteins at the plasma membrane and an increase in IEC permeability. Remarkably, glutamine supplementation in these *in vitro* models completely reversed this process, suggesting that sufficient availability of free glutamine is crucial for optimal epithelial barrier functions. These effects were mediated through enhanced AMP-activated protein kinase signaling and diminished PI3K/Akt signaling, indicating that glutamine supports intestinal barrier function via modulation of specific intracellular pathways (110, 118).

Consistent with *in vitro* studies in neonatal cells, studies in young animals also suggest a potential role of glutamine in promoting a healthy intestinal development. In rat pups and young piglets, dietary deprivation of glutamine has been reported to diminish intestinal integrity, through breakdown of epithelial junctions and shortening of microvilli (119, 120). Conversely, dietary supplementation of glutamine in young piglets has been consistently reported to increase villus height, inhibit apoptosis and boost proliferation of IECs, increase tight junction protein expression and improve epithelial barrier function (98, 121–123). In addition, glutamine is shown to protect against pathogen-induced intestinal damage *in vivo*. For instance, weaning piglets fed a glutamine-enriched diet prior to challenge with *E.coli* completely maintained villus morphology and tight junction protein expression (124, 125). Moreover, oral supplementation of glutamine prevented endotoxin-induced intestinal damage in suckling piglets (114). Consistent with the ability of glutamine to promote intestinal barrier function, glutamine supplementation is reported to prevent bacterial translocation in various adult animal models of intestinal obstruction (126–131). Whether glutamine can also prevent bacterial translocation in neonatal animals remains to be examined.

#### Impact of Glutamate on Intestinal Functions

A growing body of evidence suggests that next to glutamine also glutamate has effects on IEC growth and intestinal barrier function. A recent *in vitro* study in neonatal porcine IECs has demonstrated that supplementation of glutamate dose-dependently enhances cell proliferation (132). Moreover, this study showed that glutamate supplementation prevented oxidative stress-induced changes in IEC viability, barrier function and membrane integrity by increasing the abundance of tight junction proteins (132). The ability of glutamate to improve intestinal barrier function is also demonstrated in a study using adult human IEC lines, where glutamate addition significantly reduced phorbol-induced hyperpermeability (133). Remarkably, these effects were observed at a glutamate concentration three times lower than that present in human milk, highlighting the potency of free glutamate in human milk to exert physiological effects.

In addition to *in vitro* studies, *in vivo* studies in young animals also indicate that free glutamate can promote intestinal development. Supplementation of dietary glutamate to healthy weaning piglets led to an increase in overall intestinal health, as evidenced by higher villus height and enhanced intestinal mucosal thickness and integrity (122, 134). Furthermore, dietary glutamate dose-dependently enhanced the weight of the small intestine, increased the depth of the crypts and the lamina propria, and improved intestinal antioxidative capacities in healthy weaning piglets (99). Finally, dietary glutamate prevented mycotoxin-induced impairments in intestinal barrier function and morphology in young piglets, suggesting that free glutamate may also play a role in the prevention of intestinal damage (135).

As glutamate can be converted into glutamine by IECs, although at limited rates, the effects observed for glutamate may be attributable to the effects of glutamine. However, studies examining effects of both glutamine and glutamate demonstrated differential effects of these FAAs on functions of IECs and intestinal morphology. For instance, weaning piglets supplemented with dietary glutamine alone had higher villi than those piglets supplemented with a combination of glutamate and glutamine, whereas the combination led to the deepest crypts (136). Moreover, glutamine was observed to have protective effects against oxidant- and endotoxin-induced death of porcine neonatal IECs *in vitro*, whereas glutamate had no effect (114). This indicates that the effects of glutamate on intestinal function are not solely exerted through conversion into glutamine.

### Effects of Free Glutamine and Glutamate on Immune Cell Functions

In addition to epithelial cells, the immune cells of the GALT also play a crucial role in the prevention of neonatal allergies and infections. The immature neonatal GALT is characterized by the production of higher levels of pro-inflammatory cytokines (137, 138), whereas anti-inflammatory capacities are diminished (139). The pro-inflammatory milieu in the neonatal intestines is indicated to induce T-helper 2 (T_H_2) immune activity (140, 141). In contrast, T-helper 1 (T_H_1) immunity is highly limited and gradually develops during the postnatal period (142–144). The resulting T_H_2-dominant immune milieu is known to increase the susceptibility to allergic sensitization, whereas the minimal T_H_1 function correlates with the increased susceptibility of neonates to infections (144, 145). Thus, components in human milk with anti-inflammatory capacities, or components that enhance the development of T_H_1 immunity or suppress T_H_2 activity might contribute to the prevention of neonatal allergies and infections. Free glutamine and glutamate both have been associated with these immunomodulatory capacities, as described in detail below.

#### Impact of Glutamine on Immune Cell Functions

The importance of glutamine for the development and function of the immune system is well recognized. Although *in vitro* studies in neonatal cells are lacking, numerous *in vitro* studies in adult cells showed that various immune cells fail to develop and function without adequate glutamine availability (146). For instance, glutamine restriction impaired the growth and differentiation of B and T cells (147) and diminished antigen presentation and phagocytotic capacities of macrophages and neutrophils (148, 149). Conversely, glutamine supplementation dose-dependently enhanced phagocytotic capacities of human neutrophils *in vitro* (150, 151). Consistent with these findings, *in vivo* studies in young animals indicate that glutamine availability modifies intestinal immune cell populations. For example, dietary glutamine dose-dependently increased the number of neutrophils and macrophages in weaning piglets following an LPS-challenge (123, 152), suggestive of enhanced antimicrobial capacities. Moreover, in newly weaned piglets, dietary glutamine decreased the proportion of antigen-naïve T cells in the mesenteric lymph nodes (153), which are reported to be elevated in allergic patients and are proposed as an early life marker for future development of allergies (154, 155). Finally, dietary glutamine increased the number of IgA-secreting B cells in the small intestine of young mice (156) and enhanced intestinal levels of IgA in various weaning animals (157–161). Together, these results indicate that glutamine availability influences immune cell populations in developing intestinal tissues, which in turn may influence antimicrobial and anti-allergic immune processes.

A consistent body of evidence shows that glutamine also exhibits anti-inflammatory capacities. *In vitro* studies demonstrated that glutamine supplementation decreased the production of pro-inflammatory cytokines IL-6, IL-8, and/or TNFα, while increasing the production of anti-inflammatory/regulatory cytokine IL-10 in various activated adult human immune cells, including intra-epithelial lymphocytes (IELs), intestinal mast cells, peripheral mononuclear cells (PBMCs) and monocytes (162–165). Similar findings are reported in healthy young animals. For instance, dietary glutamine reduced levels of pro-inflammatory cytokines (including IL-1 and IL-8) while increasing levels of anti-inflammatory/regulatory cytokines (including IL-10) in the small intestine of healthy weaning piglets (123, 124, 166). Furthermore, in LPS-challenged piglets, dietary glutamine reduced intestinal expression of inflammatory markers, including Toll-like receptor-4 and the nuclear factor NF-κB, suggesting that glutamine might also have potent anti-inflammatory effects in immune-compromised conditions (114).

Glutamine has also been indicated to play a regulating role in the balance between T_H_1 and T_H_2 immunity, however, *in vitro* studies examining this aspect in neonatal immune cells are lacking. It is reported that adult murine naïve T cells are able to differentiate into T_H_2 cells under glutamine-restricted conditions, but not into functional T_H_1 cells, indicating that glutamine deprivation may favor T_H_2 differentiation (167). Conversely, supplementation of glutamine is reported to enhance T_H_1 and/or diminish T_H_2 responses of various activated adult immune cells *in vitro*. For instance, glutamine increased the production of T_H_1 cytokines IL-2 and IFNy by activated murine IELs and by human lymphocytes and PBMCs, while T_H_2 cytokines were unaltered (168–171). In activated human intestinal mast cells, glutamine did not alter the release of T_H_1 chemokines, but reduced the release of T_H_2 chemokine ligand 2 and leukotriene C4, which are both involved in the pathogenesis of various allergic diseases (164, 172). Although data are limited, *in vivo* studies in young animals also suggest a regulating role of glutamine in the T_H_1/T_H_2 immune balance. In young mice, dietary glutamine increased the expression of IL-2 and the IL-2 receptor by lymphocytes, indicative of increased activity of and responsiveness to T_H_1 stimuli (173). Moreover, dietary glutamine in healthy weaning piglets lowered the production of T_H_2 cytokine IL-4 and increased the IFNγ/IL-4 ratio in mesenteric lymph node cells (153). Finally, in weaning rabbits, dietary glutamine upregulated IL-2 and IL-10 expression by IELs, while inhibiting expression of IL-6, an inducer of T_H_2 differentiation of naïve T cells (174, 175). Although further confirmation in neonatal animals is critical, these data may indicate that glutamine plays a role in promoting a more balanced T_H_1/T_H_2 immune system in the neonatal period.

#### Impact of Glutamate on Immune Cell Functions

Despite dietary glutamate being almost completely used in intestinal tissues, studies investigating the effects of glutamate on intestinal immune cells are lacking. Yet, receptors for glutamate are found on a variety of immune cells, including lymphocytes and dendritic cells, suggesting that glutamate has a role in immune cell functioning (176). Studies using adult human peripheral T cells demonstrated that glutamate at low concentrations (<100 μM) dose-dependently increases the proliferative response of T cells to various stimuli (176, 177). At higher concentrations (>1 mM), however, this effect reversed, indicating that glutamate tends to have immunosuppressive properties at higher concentrations (176, 178). Accordingly, it is postulated that the high glutamate concentration in the intestinal microenvironment, which may reach the millimolar range, could prevent inappropriate responses to dietary antigens by exerting immunosuppressive effects on intestinal T cells (178).

Besides regulating T cell proliferation, glutamate availability is also indicated to influence the T_H_2 and T_H_1 cytokine production by T cells. Glutamate is released by dendritic cells during T cell interaction, where it has dual roles (179). In cases of non-specific antigen presentation, glutamate inhibits T cell activation. However, upon specific antigen presentation glutamate stimulates T cell proliferation and the production of IL-2, IFNγ and IL-10, thereby promoting a T_H_1 response (179). This latter process depends on glutamate released from dendritic cells, but also on extracellular glutamate concentrations, suggesting that this process could be influenced by dietary glutamate (179). Accordingly, it is reported that glutamate supplementation of up to 1-2 mM enhanced IFNy and IL-10 secretion by activated adult human peripheral T cells *in vitro*, whereas secretion of T_H_2 cytokines IL-4 and IL-5 was unaffected (180). When supplied at even higher concentrations (>5 mM), however, glutamate inhibited IFNy and IL-10 secretion by these cells. Unfortunately, *in vitro* studies in neonatal cells and *in vivo* studies investigating the effects of glutamate on immune cell functions are lacking. Nevertheless, the findings in adult immune cells suggest an immunoregulating role for glutamate, with effects that are highly dependent on the context and the concentration. At concentrations present in human milk, glutamate could be involved in promoting T_H_1 immunity and subsequently in reducing the susceptibility to allergic sensitization, although this remains speculative due the lack of evidence in neonatal cells or animals.

### Effects of Free Glutamine and Glutamate on the Intestinal Microbiota

Accumulating evidence indicates that the gut microbiota plays a vital role in tolerance induction to dietary antigens (181–183). Accordingly, clinical studies have provided evidence for a link between the microbiota composition in the neonatal period and the development of allergic diseases. It is reported that a higher intestinal bacterial diversity in early life is associated with a lower risk of developing various allergic diseases, including food allergy (184–187). Moreover, infants with an increased colonization of *Firmicutes* and a decreased colonization of *Bacteroidetes* (corresponding to an increased *Firmicutes*-to-*Bacteroidetes* ratio), or a decreased colonization of *Proteobacteria* and *Bifidobacteria* are shown to be at increased risk of developing food allergies (188–191). Mechanisms by which gut microbes modify the susceptibility to allergies are poorly understood but may involve specific modulation of T_H_2 and T_H_1 immunity (192, 193). The colonization of intestinal microbiota is far from complete at birth and is influenced by various environmental factors, including breastfeeding duration (189). Thus, human milk components that shape the neonatal gut microbiota composition may play an active part in modifying the susceptibility to allergic sensitization. Although data are limited, several studies have shown that glutamine and glutamate can modulate the abundance of gut bacteria that have been associated with the protection against allergic diseases.

#### Impact of Glutamine on the Gut Microbiota Composition

The ability of dietary glutamine to modify the microbiota composition is shown in various young animals. A study in weaning mice demonstrated that dietary glutamine decreased the content of *Firmicutes* in the jejunum and ileum, and decreased the *Firmicutes*-to-*Bacteroidetes* ratio in the ileum (194). Similar findings are reported in studies in adult pigs and human (195, 196). In weaning rabbits, dietary glutamine specifically reduced the presence of *Clostridium spp*. in the ileum, of which colonization in early life has been associated with increased risk of allergic diseases (197, 198). Finally, a glutamine-enriched diet is also shown to increase the abundance of beneficial *Bifidobacteria* in the jejunum of healthy weaned mice (194), and to decrease potentially harmful microorganisms in adult pigs (196). The mechanisms underlying the effects of glutamine on the gut microbiota composition are poorly understood. It is postulated that glutamine supplementation regulates utilization and metabolism of a variety of AAs in a niche-specific manner, affecting the activity and number of specific microbes (157, 199).

#### Impact of Glutamate on the Gut Microbiota Composition

To our knowledge, only two animal studies examined the effects of dietary glutamate on the intestinal microbiota composition to date, both of which used animals in their post-weaning phase. It has been reported that dietary glutamate markedly enhanced the bacterial diversity in the intestinal flora of healthy post-weaning pigs (200). Moreover, the glutamate-enriched diet decreased the *Firmicutes*-to-*Bacteroidetes* ratio in the ileum, although this effect was only seen when given in combination with a high fat diet and was not observed in other intestinal sections. Perhaps more interestingly, dietary glutamate specifically promoted the colonization of *prausnitzii* and *Faecalibacterium prausnitzii* in post-weaning pigs (200, 201). The colonization of *Roseburia* in early life has been positively associated with the acquisition of tolerance to cow's milk (202), and *Faecalibacterium prausnitzii* is indicated to play a role in the prevention of food allergy (203–205). These intestinal microbes are some of the main producers of the short-chain fatty acids butyrate and propionate. Accordingly, a glutamate-enriched diet significantly increased colonic concentrations of these fatty acids in adult pigs (206). Butyrate and propionate both have been associated with the prevention of various allergic diseases and, consequently, high faecal levels of these fatty acids in early life have been associated with a decreased risk of developing atopy (207–209).

## Concluding Remarks

Research indicates that breastfeeding during the first months of life provides protection against immune-related conditions in neonates and later in life. These conditions include gastrointestinal infections and several allergic diseases including food allergy. It is indicated that the transfer of specific immunomodulating components, such as bioactive proteins, from mother to infant through human milk contributes to this protective effect. In addition to proteins, human milk contains FAAs, which have unique characteristics. They are more readily absorbed than protein-derived AAs and can be recognized by specific receptors on various cells. Moreover, whereas protein-bound AAs decrease during the lactation period in a non-AA-specific manner, temporal changes of FAAs in human milk are highly AA-specific. These dynamics in FAA levels are globally consistent and thus seemingly independent of ethnicity, demographics and maternal diet. This suggests that selective FAA transport occurs in the mammary gland, that FAA levels in human milk are strictly regulated and, consequently, that FAAs are likely to be of physiological relevance in the developing infant.

With regards to individual FAAs in human milk, free glutamine and glutamate display particularly remarkable characteristics. They account for almost 70% of the FAA content in human milk, they both drastically increase in the first 3 months of lactation and their levels have been shown to positively correlate with infant growth, suggestive of important functions in the developing neonate. In neonates, dietary glutamine and glutamate are mainly used by the intestines. Remarkably, studies in neonatal immune cells and young animals demonstrate that these FAAs can have a wide range of effects on cells in developing intestines, also at concentrations similar to their levels in human milk. In short, they are reported to increase the growth of intestinal epithelial cells, enhance intestinal barrier function, influence immune cell development and populations in the gut-associated lymphoid tissue, exert anti-inflammatory and potentially T_H_1 promoting and/or T_H_2 inhibiting effects on various intestinal immune cells, and modify the abundance of gut microbiota that might play a role in allergic sensitization (Figure 1). Together, these effects could potentially support neonates in the protection against allergic sensitization and infections.

All together, the findings described in this review warrant further research into the contribution of free glutamine and glutamate in human milk to the protection against neonatal allergies and infections. Levels of free glutamine and glutamate, in addition to that of other bioactive factors that could influence early life immune development, are considerably higher in human milk than in standard infant milk formulas, leading to significant differences in the intake of these FAAs between breastfed and formula-fed children (210–212). As many of the effects of glutamine and glutamate described in this review were concentration-dependent, future studies should address whether this differential intake contributes to the differential occurrence in immune-related conditions between formula-fed and breastfed children.

## Author Contributions

JS, AH, and SW: conceptualization and literature searches. AH, SW, and JG: supervision. JS: writing original draft. JS, AH, SW, and JG: review and editing. All authors read and approved the manuscript.

## Conflict of Interest

SW is current employee of Danone Nutricia Research. JG is part-time employee of Danone Nutricia Research and Utrecht University. The remaining authors declare that the research was conducted in the absence of any commercial or financial relationships that could be construed as a potential conflict of interest.
